# Circulating MicroRNAs as Non-Invasive Biomarkers for Early Detection of Non-Small-Cell Lung Cancer

**DOI:** 10.1371/journal.pone.0125026

**Published:** 2015-05-12

**Authors:** Magdalena B. Wozniak, Ghislaine Scelo, David C. Muller, Anush Mukeria, David Zaridze, Paul Brennan

**Affiliations:** 1 Genetic Epidemiology Group, International Agency for Research on Cancer (WHO-IARC), Lyon, France; 2 Institute of Carcinogenesis, N. N. Blokhin Cancer Research Centre, Moscow, Russia; Deutsches Krebsforschungszentrum, GERMANY

## Abstract

**Background:**

Detection of lung cancer at an early stage by sensitive screening tests could be an important strategy to improving prognosis. Our objective was to identify a panel of circulating microRNAs in plasma that will contribute to early detection of lung cancer.

**Material and Methods:**

Plasma samples from 100 early stage (I to IIIA) non–small-cell lung cancer (NSCLC) patients and 100 non-cancer controls were screened for 754 circulating microRNAs via qRT-PCR, using TaqMan MicroRNA Arrays. Logistic regression with a lasso penalty was used to select a panel of microRNAs that discriminate between cases and controls. Internal validation of model discrimination was conducted by calculating the bootstrap optimism-corrected AUC for the selected model.

**Results:**

We identified a panel of 24 microRNAs with optimum classification performance. The combination of these 24 microRNAs alone could discriminate lung cancer cases from non-cancer controls with an AUC of 0.92 (95% CI: 0.87-0.95). This classification improved to an AUC of 0.94 (95% CI: 0.90-0.97) following addition of sex, age and smoking status to the model. Internal validation of the model suggests that the discriminatory power of the panel will be high when applied to independent samples with a corrected AUC of 0.78 for the 24-miRNA panel alone.

**Conclusion:**

Our 24-microRNA predictor improves lung cancer prediction beyond that of known risk factors.

## Introduction

Lung cancer is the most common cause of cancer death worldwide. In 2012, 1.82 million new cases, and 1.59 million deaths due to lung cancer were recorded, representing 13% of all cancer cases and 19% of all cancer deaths respectively [1]. Non-small cell lung cancer (NSCLC) accounts for approximately 80–85% of all lung cancer cases and comprises primarily two histological types: adenocarcinoma (AC) and squamous cell carcinoma (SCC). In spite of advances in therapy, an overall 5-year survival rate of only 16% [2] is mostly due to late stage at diagnosis. Detection of lung cancer at an early stage by sensitive screening tests could be an important strategy to improve lung cancer prognosis.

The National Lung Screening Trial (NLST) using low-dose helical computed tomography (LDCT) in high-risk individuals demonstrates that a 20% reduction in lung cancer-specific mortality and a 6.7% reduction in all-cause mortality [3] can be achieved. Nevertheless, high false-positive rates of NLST [4], costs, and potential harms from radiation exposure highlight the need for simpler, non-invasive and more accessible methodologies for effective early cancer detection as complementary biomarkers.

MicroRNAs (miRNAs) are a group of small (~22-nucleotides long) non-coding, single-stranded RNAs that regulate gene expression post-transcriptionally. Aberrations in miRNA expression levels have been found in relation to oncogenesis and tumour metastasis [5], including NSCLC. More than 2500 human miRNAs sequences are currently known [6]. Several studies have shown that serum and plasma miRNAs (called circulating miRNAs) present great promise as novel non-invasive biomarkers for the early diagnosis of various cancers due to their ease of access, and long term stability [7,8]. In lung cancer, several miRNA expression profiles have been identified with remarkably high predictive values including a 34-miRNA diagnostic signature with an AUC of 0.89 [9], a 10-miRNA panel with an AUC of 0.97 in serum as well as 16-miRNA ratios as a signature of risk (AUC: 0.85) and diagnosis (AUC: 0.88) [10,11] in plasma samples of NSCLC patients.

Previous studies have shown substantial inconsistencies although these were based on limited sample sizes or used candidate miRNA approaches. Additionally, a level of controversy exists in that miRNAs identified in the blood merely indicate changes in blood cells secondary to an overall poor health condition and are not specific to the presence of cancer in a target organ. Although the measurement of miRNA expression in plasma or serum has been postulated as a promising approach in diagnosing lung cancer, the concept requires further investigations to demonstrate its potential use as a clinical application. There is no strong evidence as to whether serum or plasma is superior for miRNA evaluation, however, recent reports suggest that plasma may be the preferred sample choice given that RNA released during the coagulation process may alter the composition of circulating miRNAs in serum samples [12].

Here, we present a genome-wide miRNA expression screen in plasma samples from 100 NSCLC cases and 100 controls in which we determined a miRNA signature with a high diagnostic value that was identified through stringent statistical approaches.

## Results

### Study population

The characteristics of 200 study participants, including 65 patients with lung SCC, 35 patients with lung AC and 100 controls are presented in Table 1. There was a difference in sex (more men among the cases), age at interview (cases were on average 1.5 years older than controls), smoking status (15% more current smokers among cases) and alcohol drinking status (cases included 12% more former alcohol consumers). Lung cancer patients were of an early IA—IIIA stage. The mean follow-up time was 2.48 years. During this follow-up period, 64 of the 100 cases died and 36 were still alive at censoring.

**Table 1:** Select baseline characteristics of the study population (lung cancer cases and controls) recruited in the IARC case-control study (Moscow, Russia, 2006–2012).

Characteristics	Cases	Controls	p value^a^
	N = 100	N = 100	
**Sex**			0.010
Female	14	29	
Male	86	71	
**Age at interview**, mean (SD)	62.6 (7.5)	60.1 (9.7)	0.037
**Smoking status**			<0.001
Never	11	40	
Former	27	12	
Current	62	48	
**Pack-years** (among smokers only) mean (SD)	47.8 (22.5)	29.8 (15.2)	<0.001
**Alcohol drinking status**			0.029
Never	23	36	
Former	23	11	
Current	54	53	
**Body mass index (BMI)** [kg/m2] mean (SD)	26.2 (4)	27.3 (4.7)	0.058
**Histological type**			
Squamous cell carcinoma	65	———-	
Adenocarcinoma	35	———-	
**Clinical stage**			
IA	16	———-	
IB	33	———-	
IIA	6	———-	
IIB	15	———-	
IIIA	30		
**Years of follow-up**, mean (SD)	2.48 (1.69)	———-	
**Vital status at the end of follow-up**			
Dead	64	———-	
Alive	36	———-	

^a^ p value calculated using χ^2^ test for categorical variables and Student’s t-test for continuous variables.

### Micro-RNA profiles in plasma

**Fig 1:**
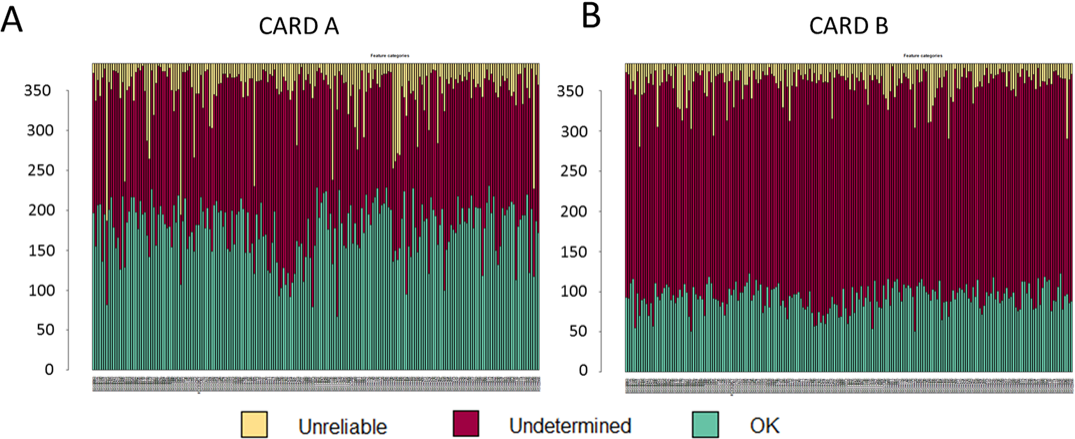
MiRNA detected in plasma samples of 100 patients with lung cancer vs 100 controls on TaqMan microRNA CARD A and CARD B, respectively in the IARC case-control study (2006–2012).

To evaluate whether specific miRNA signatures are detectable in plasma samples of patients with early stages of NSCLC, we performed a high-throughput screen of 754 miRNAs using TaqMan Human MicroRNA Arrays. On average 235 and 115 miRNAs were detected on Card A and Card B, respectively (present in at least 80 out of 200 samples in either cases or controls) (Fig 1). The miRNA expression profiles in plasma were quantile normalized and subjected to differential expression analyses between NSCLC samples and controls. Of 350 detected miRNAs in plasma, 61 miRNAs were found to be significantly differentially expressed between lung cancer cases and controls (35 on Card A, 26 on Card B) including 33 upregulated and 28 downregulated miRNAs (p-value < 0.05). These comprised 21 miRNAs differentially expressed with significant adjusted p-value corrected for multiple testing (Table 2). Despite the significant differential expression, supervised hierarchical clustering analysis showed that this set of 61 miRNAs was not able to clearly distinguish between lung cancer cases and controls (S1 Fig).

**Table 2:** Complete list of significant differentially expressed miRNA (p-value < 0.05) in lung cancer patients as compared with controls in the IARC case-control study (Moscow, Russia, 2006–2012)—global miRNA expression profiling using TaqMan Human MicroRNA Array A + B Card Set (v3.0).

miRNA	p-value^a^	adj. p-value^b^	FC	TLDA card
miR-340#-002259	5.99E-06	0.0006	2.92	B
miR-218-000521	6.68E-06	0.0010	0.29	A
miR-450b-5p-002207	1.04E-05	0.0010	0.31	A
miR-566-001533	0.0003	0.0144	0.30	B
miR-661-001606	0.0005	0.0160	0.36	B
miR-200c-002300	0.0004	0.0212	2.65	A
miR-323-3p-002227	0.0005	0.0212	0.55	A
miR-642-001592	0.0007	0.0212	0.43	A
miR-28-000411	0.0007	0.0212	1.91	A
miR-1290-002863	0.0011	0.0264	0.54	B
miR-590-3P-002677	0.0012	0.0264	2.29	B
miR-485-3p-001277	0.0012	0.0331	0.56	A
miR-22#-002301	0.0021	0.0364	2.18	B
miR-191#-002678	0.0027	0.0402	2.88	B
miR-26b#-002444	0.0039	0.0442	3.08	B
miR-34b-002102	0.0042	0.0442	0.39	B
miR-425#-002302	0.0043	0.0442	2.24	B
miR-766-001986	0.0046	0.0442	1.65	B
miR-203-000507	0.0019	0.0445	0.37	A
miR-193a-5p-002281	0.0024	0.0474	0.49	A
miR-25-000403	0.0025	0.0474	0.62	A
miR-517b-001152	0.0033	0.0515	0.28	A
miR-487a-001279	0.0033	0.0515	0.40	A
miR-1275-002840	0.0065	0.0571	0.42	B
miR-122-002245	0.0041	0.0591	0.53	A
miR-378-000567	0.0078	0.0638	0.43	B
miR-146a-000468	0.0059	0.0775	1.78	A
miR-483-5p-002338	0.0065	0.0775	0.52	A
miR-484-001821	0.0066	0.0775	1.90	A
miR-93#-002139	0.0113	0.0857	1.73	B
miR-652-002352	0.0103	0.1064	1.70	A
miR-708-002341	0.0105	0.1064	0.38	A
miR-339-3p-002184	0.0107	0.1064	1.64	A
miR-200a-000502	0.0118	0.1086	0.46	A
miR-182-002334	0.0121	0.1086	0.56	A
miR-1243-002854	0.0176	0.1241	0.37	B
let-7f-000382	0.0150	0.1278	3.13	A
miR-151-3p-002254	0.0215	0.1390	1.63	B
miR-1271-002779	0.0235	0.1390	1.75	B
miR-454#-001996	0.0236	0.1390	1.84	B
let-7b-002619	0.0188	0.1488	1.33	A
miR-140-001187	0.0190	0.1488	1.52	A
miR-26a-000405	0.0217	0.1632	1.36	A
miR-155-002623	0.0226	0.1633	1.54	A
miR-645-001597	0.0305	0.1702	0.56	B
miR-191-002299	0.0249	0.1728	1.35	A
miR-221-000524	0.0261	0.1728	1.46	A
miR-18a-002422	0.0267	0.1728	1.75	A
miR-1285-002822	0.0332	0.1757	0.34	B
miR-30d#-002305	0.0385	0.1802	1.90	B
miR-130b#-002114	0.0407	0.1802	2.14	B
miR-7#-001338	0.0418	0.1802	1.37	B
miR-30a-3p-000416	0.0425	0.1802	1.57	B
miR-335#-002185	0.0425	0.1802	1.64	B
miR-1233-002768	0.0452	0.1842	0.46	B
miR-495-001663	0.0315	0.1953	0.44	A
miR-758-001990	0.0322	0.1953	0.56	A
miR-223-002295	0.0425	0.2497	1.51	A
miR-411-001610	0.0479	0.2676	0.60	A
miR-19b-000396	0.0493	0.2676	1.54	A
miR-26b-000407	0.0498	0.2676	1.48	A

^a^ Limma analysis non-adjusted p-value.

^b^ p-value corrected for multiple testing (Benjamini-Holm method).

FC: fold change (> 1 increased; < 1 decreased expression in lung cancer patients vs. non-cancer controls).

### Prediction models

**Fig 2:**
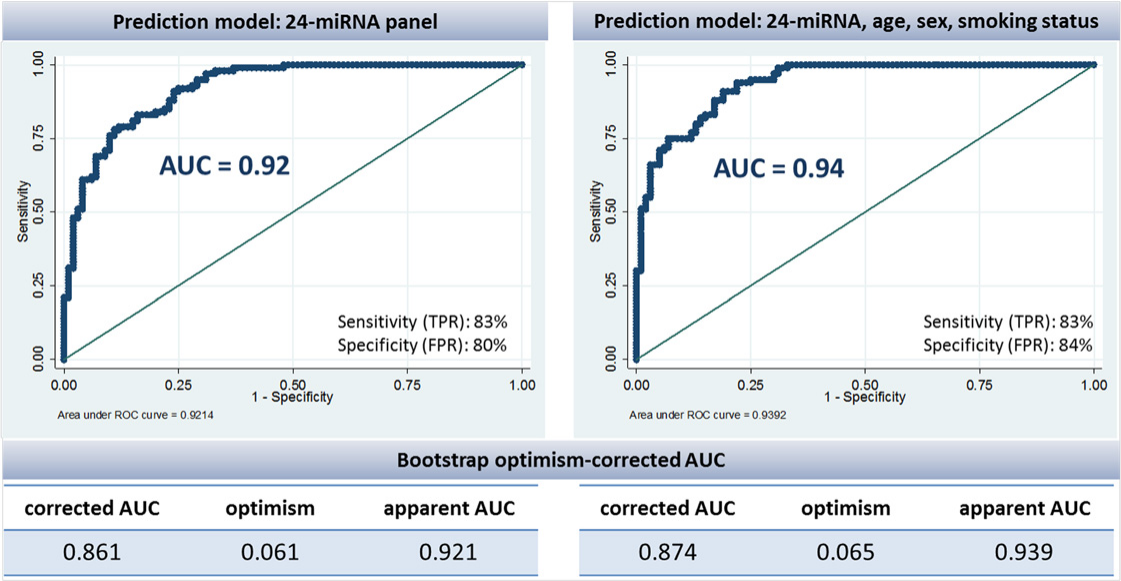
Evaluation of the performance of the 24-miRNA classifier (left) and a classifier including the 24-miRNA panel, age at recruitment, sex and smoking status (right) assessed using area under Receiver Operating Characteristics (ROC) curves (AUCs) in the IARC case-control study (2006–2012).

**Fig 3:**
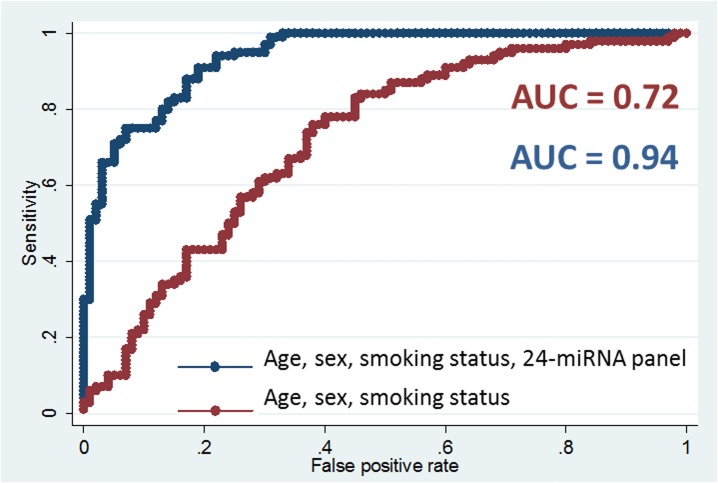
Evaluation of the performance of the model including age at recruitment, sex and smoking status (red) and a classifier including 24-miRNA panel, age at recruitment, sex and smoking status (blue) assessed using the area under Receiver Operating Characteristics (ROC) curves (AUCs) in the IARC case-control study (2006–2012).

To select a panel of miRNAs discriminating between cases and controls, a logistic regression model with lasso penalty was fitted. This analysis identified a panel of 24 plasma miRNAs with an optimum classification performance (S2 and S3 Figs). Table 3 shows the complete list of selected miRNAs within the panel. Multiple logistic regression analysis showed that combination of the 24 miRNAs alone could discriminate lung cancer cases from controls with very high AUC of 0.92 (95%CI: 0.87–0.95). In the logistic model including the 24-miRNA panel and adjusted for main lung cancer risk factors, namely sex, age at interview and smoking status, the classification improved to an AUC of 0.94 (95%CI: 0.90–0.97) (Fig 2). The improvement in the magnitude of discrimination attributable to the 24-miRNA panel is substantial compared with that of the main lung cancer risk factors alone. The AUCs for the full logistic models with and without the 24-miRNA panel were 0.94 (95%CI: 0.90–0.97) and 0.72 (95%CI: 0.65–0.78), respectively (Fig 3). The predictive value was of similar magnitude (AUC: 0.95; 95%CI: 0.91–0.98) when replacing the categorical smoking status variable by the continuous pack-years variable. Notably, even though derived from both histological types, the 24-miRNA panel performed equally well in both ACs (AUC: 0.94) and SCCs (AUC: 0.96). Similarly, the predictor performed well for cancers of all stages (IA-IIIA), with an AUC of 0.96 for stage I (IA and IB; n = 49) patients, 0.98 for stage II (stage IIA and IIB; n = 21) patients and 0.97 for stage IIIA (n = 30) patients (data not shown).

**Table 3:** Logistic regression prediction model with the 24-microRNA panel for patients with lung cancer vs controls in the IARC case-control study (Moscow, Russia, 2006–2012).

Variable	OR^a^ (95%CI)	P value^a^	OR^b^ (95%CI)	P value^b^
Sex	———-	———-	1.80 (0.37–8.74)	0.466
Age at interview	———-	———-	1.02 (0.95–1.09)	0.578
Smoking status		———-		0.003
Never	———-		Reference	
Former	———-		24.46 (3.33–179.8)	
Current	———-		19.09 (3.19–114.2)	
hsa-let-7c-000379	0.75 (0.56–1.00)	0.051	0.71 (0.50–0.99)	0.043
hsa-miR-122-002245	1.16 (0.93–1.46)	0.196	1.17 (0.89–1.53)	0.273
hsa-miR-182-002334	1.32 (1.04–1.67)	0.021	1.36 (1.03–1.79)	0.029
hsa-miR-193a-5p-002281	1.14 (0.92–1.42)	0.220	1.07 (0.85–1.35)	0.538
hsa-miR-200c-002300	0.81 (0.68–0.98)	0.029	0.89 (0.73–1.10)	0.274
hsa-miR-203-000507	1.12 (0.95–1.32)	0.168	1.10 (0.92–1.31)	0.291
hsa-miR-218-000521	1.24 (1.02–1.52)	0.033	1.13 (0.91–1.39)	0.272
hsa-miR-155-002623	0.84 (0.64–1.00)	0.202	0.84 (0.63–1.12)	0.238
hsa-let-7b-002619	0.95 (0.61–1.48)	0.826	0.96 (0.58–1.58)	0.878
hsa-miR-411-001610	1.37 (1.12–1.68)	0.002	1.41 (1.13–1.75)	0.002
hsa-miR-450b-5p-002207	1.37 (1.11–1.70)	0.003	1.52 (1.19–1.94)	0.001
hsa-miR-485-3p-001277	1.40 (0.97–2.01)	0.072	1.34 (0.91–1.99)	0.141
hsa-miR-519a-002415	1.22 (1.05–1.42)	0.011	1.20 (1.01–1.42)	0.044
hsa-miR-642-001592	1.13 (0.93–1.37)	0.221	1.29 (1.03–1.62)	0.026
hsa-miR-517b-001152	1.07 (0.95–1.21)	0.260	1.10 (0.96–1.27)	0.169
hsa-miR-520f-001120	1.06 (0.99–1.13)	0.113	1.10 (1.01–1.18)	0.021
hsa-miR-206-000510	0.85 (0.74–0.99)	0.033	0.90 (0.76–1.06)	0.203
hsa-miR-566-001533	1.10 (0.95–1.26)	0.197	1.02 (0.86–1.21)	0.817
hsa-miR-661-001606	1.10 (0.94–1.30)	0.240	1.15 (0.96–1.39)	0.130
hsa-miR-340#-002259	0.74 (0.57–0.97)	0.030	0.71 (0.52–0.96)	0.027
hsa-miR-1243-002854	1.09 (0.97–1.22)	0.139	1.10 (0.97–1.26)	0.131
hsa-miR-720-002895	1.95 (1.39–2.73)	<0.001	1.89 (1.28–2.80)	0.001
hsa-miR-543-002376	1.11 (0.95–1.30)	0.170	1.15 (0.97–1.36)	0.112
hsa-miR-1267-002885	0.94 (0.88–1.00)	0.048	0.95 (0.88–1.01)	0.114

^a^ Model containing the 24-miRNA panel (continuous, normalized Ct values).

^b^ Model containing sex (Male, Female), age at interview (continuous), smoking status (Never, Former, Current) and the 24-miRNA panel (continuous, normalized Ct values).

Abbreviations: OR, odds ratio; CI, confidence interval.

Internal validation of the selected 24-miRNA panel model (i.e., validation accounting for variability due to parameter estimation) using the bootstrap optimism corrected AUC suggests that the discriminatory power of the panel will be high when applied to independent samples (corrected AUC of 0.86 for the 24-miRNA panel alone, 0.87 for the model including sex, age and smoking status and 0.89 for the model with pack-years variable). The bootstrap optimism corrected AUC which took into account the entire miRNA classifier selection process (penalized Lasso logistic regression) was 0.78.

## Discussion

Despite extensive advancements of imaging and combined treatment modalities, the 5-year survival rate of lung cancer has improved only marginally over recent decades [2]. A non-invasive, biomarker-driven stratification of early-stage lung cancer could therefore complement LDCT screening and improve therapy management. We have developed a panel of 24 plasma miRNAs capable of discriminating early stage NSCLC cases from controls with a high AUC of 0.92 after screening 754 miRNAs in 100 NSCLC patients and 100 non-cancer individuals. This set of miRNAs was identified through very stringent statistical methods. To our knowledge this is also one of the largest exploratory studies of miRNA in plasma. Tests such as these demonstrate several advantages in the clinical setting including no requirement for invasive sample collection (“liquid biopsy”), low cost when compared to imaging techniques, straightforward laboratory procedures and inclusion of a panel of biomarkers instead of a single miRNA.

To date, several studies have reported miRNA profiles in plasma and serum developed for the diagnosis of NSCLCs [9–11,13–16]. Despite very promising results these studies have shown a rather small overlap between identified miRNA signatures, and were based on limited sample sizes/pools of samples which did not allow for assessment of the contribution by a single patient to the genetic pool, or used a candidate miRNA approach for discovery of the miRNA panel. The variation in pre-analytical factors, such as sample preparation procedures, and different normalization strategies makes comparison between studies difficult. Also the differences in miRNA profiles between serum and plasma [14] may account for some of the lack of correlation of miRNA expression levels between previous studies. Lastly, the described signatures could differ because of the inherent multitude of miRNA targets and their potential redundancy. Given that our study evaluated the highest number of miRNA profiles, we were able to assess the predictive value of previously reported miRNA signatures in our data. Interestingly, despite small overlap of miRNAs between predictors, different population of patients, sample processing, extraction protocols and normalization procedures used when compared to earlier studies [9,10,13], a relatively high predictive value (AUC: 0.68–0.78) of these miRNA panels was observed in our study (S1–S4 Tables, S4 Fig), thus highlighting the potential of circulating miRNAs as biomarkers for lung cancer detection. In our data, the best discrimination between NSCLC cases and controls was found for the 34-miRNA signature reported by Bianchi and colleagues [9] showing an AUC of 0.78 (95%CI: 0.72–0.84). Lower predictive value was observed when using the signature of risk (developed in pre-diagnostic samples) and diagnosis (developed in case-control study) described by Boeri and colleagues [10] and recently validated by the same group [11] showing an AUC of 0.71 (95%CI: 0.65–0.78) and AUC of 0.70 (95%CI: 0.63–0.76), respectively. Finally, the 10-miRNA panel defined by Chen and colleagues yielded an AUC of 0.68 (95%CI: 0.61–0.74) when fitted using our data [13].

Previous reports raised the question whether miRNAs found in circulation originate from tumours. In theory, miRNA in plasma or serum can originate from the tumour or from inflammatory host responses. In the absence of miRNA data from tumour tissue in our set of samples we compared a panel of 24 miRNAs in our predictor to differentially expressed miRNA from AC and SCC of the lung obtained via analysis of the Cancer Genome Atlas (TCGA) miRNA sequencing data (https://tcga-data.nci.nih.gov/tcga/). Twelve of the miRNA included in our predictor were also altered in TCGA data in either histology. However, the direction of association was consistent only for let-7c and miR-218. This suggests that a predictive role of plasma miRNAs is independent from tissue in line with previous findings of Boeri and colleagues [10].

Recent studies have also suggested that significant variations in abundance of microRNA biomarkers reported in the literature might be a result of the inclusion of haemolyzed samples [17–19]. To minimize the effect of haemolysis, the samples used in our study followed standardized protocols and were processed within 2 hours from the time of blood collection. In addition, a QC step was implemented to assess potential haemolysis by evaluation of expression levels of 10 previously reported haemolysis-related miRNA, including miR-451, miRNA miR-16, miR-15b, miR-486-3p, miR-532-3p, miR-886-5p, miR-636, miR-1255B, RNU48 and miR-92a [17,19] among all miRNAs detected in our study. We did not observe significant differences in the haemolysis-related miRNA between lung cancer cases and controls in our series except for miR-15b (p-value = 0.024) (S5 Table). However, alterations of miR-15b have been previously reported in tumour tissues [20,21] and miR-15b has been proposed as a serum biomarker for detection of NSCLC[22]. Moreover, none of the haemolysis-related miRNAs were present in our 24-miRNA predictor.

Our study has several noteworthy strengths, including: careful selection of patients with all clinical data available, standardized and uniform processing of blood samples within 2 hours from blood collection, ultracentrifugation step following defreezing to remove cryoprecipitates and cell debris, large sample size, large number of miRNAs analyzed and very rigorous statistical assessment of the miRNA predictor. Known predictors of lung cancer were forced into models in the study regardless of statistical significance to truly test the added incremental value of our 24-miRNA panel (Fig 3).

A limitation is that, due to the case-control design of our study, blood samples were collected at the time of diagnosis. To partially address this constraint we did restrict our analysis to early stage NSCLC. Also our study lacks an external validation series. To tackle this concern we performed internal validation using a bootstrapping method which indicated that the predictive value of the panel will remain high when applied to an independent series of samples.

The small size of mature miRNAs and their sequence homology to precursor miRNA requires sensitive methods for quantitative analysis. We used the current “gold standard” method for measurement of circulating miRNAs. TaqMan MiRNA Cards use a target-specific, stem-loop reverse transcription primer to address the challenge of the short length [23]. Despite the high accuracy and specificity of the qRT-PCR technique, each miRNA expression level measured can be influenced by both systematic experimental bias and technical variations including differences in sample procurement, stabilization, RNA extraction, and sample differences. As a result, data normalization is critical to minimize “noise” and obtain biologically meaningful data and to develop miRNA-based biomarkers. In the absence of stably expressed endogenous circulating miRNAs to function as normalization controls, and to verify the sensitivity of our results several normalization strategies were tested in our study, including quantile normalization, rank normalization, geometric mean normalization, normalization to endogenous U6snRNA control and to the ath-miR-159a spike-in exogenous control. Based on exploratory data analysis plots, instability of endogenous control, difference in abundance of spike-in vs sample miRNA, we decided to quantile normalization was chosen for the analysis. In addition, to reduce confounding from technical variation such as plate-to-plate variation and variation due to purification, we distributed samples such that diagnostic variables were balanced with respect to day of analysis or plate number and randomized within each day and plate.

In summary, our study demonstrates that the 24-miRNA panel is significantly and independently associated with lung cancer following analysis of a liquid biopsy and adds to lung cancer prediction beyond that contributed by established risk factors. Our study should therefore be seen as an exploratory study providing a strong and highly predictive miRNA panel identified through rigorous statistical approaches for further validation in well-designed large prospective cohorts and screening trials. If current findings are validated, it is expected to make important contributions to clinical and public health practice and may lead to more efficient lung cancer screening by improving enrolment criteria for identifying those who would benefit from further screening.

## Patients and Methods

### Study population

Lung cancer patients and controls were recruited through an IARC case-control study coordinated in Moscow from 2006 to 2012. Cases were incident cancer patients collected from the Russian N.N.Blokhin Cancer Research Centre and Moscow City Clinical Oncology Dispensary serving Moscow and the surrounding regions. Controls were recruited from individuals visiting two Moscow general hospitals for disorders unrelated to lung cancer and to associated risk factors. All study participants provided written informed consent and were interviewed.

Peripheral blood was collected in EDTA tubes at the time of interview and processed as rapidly as possible (generally within 2 hours). For cases, blood draw was performed before surgery and any adjuvant treatment. Plasma samples were isolated by centrifugation of whole blood at 2000xg for 10 minutes at room temperature. Samples were stored at −80°C. All specimens were obtained in accordance with the declaration of Helsinki guidelines and were approved by the local Institutional Review Board and the IARC Ethics Committee. A total of 100 lung cancer cases and 100 controls were included (Table 1).

### RNA isolation

After thawing, plasma samples were centrifuged at 16,000 x g for 5 minutes to remove cryoprecipitates and cell debris. Total RNA was isolated from 300μL of plasma using NucleoSpin miRNA Plasma kit (Macherey-Nagel, Düren, Germany) according to the manufacturer’s protocol with Proteinase K digest, addition of 2μg of glycogen carrier and DNAse digest steps. All samples were spiked-in with 10pmol of *Arabidopsis thaliana* synthetic miR-159a (synthesized by Eurofins MWG Operon, Ebersberg, Germany) to control for variations in the RNA preparation step. Purified RNA was kept at −80°C before being used for reverse transcription.

### Profiling by TaqMan Human MicroRNA Arrays

Expression levels of 754 miRNAs (Sanger miRBase v14) were quantified using the TaqMan Human MicroRNA Array A + B Card Set v3.0 (Applied Biosystems, Foster City, CA) as per the manufacturer's instructions (including pre-amplification) (S1 Methods). Quantitative miRNAs expression data were acquired by ABI 7900HT SDS software v2.4. Cycle threshold (Ct, cycle in which there is the first detectable significant increase in fluorescence) values were set using ExpressionSuite software (Applied Biosystems) on the first 60 samples (automatic baseline and threshold) and these thresholds for Ct were used for the remaining series. The TaqMan Human MicroRNA Array experiments are MIAME compliant and have been deposited at the NCBI Gene Expression Omnibus (GEO) database (http://www.ncbi.nlm.nih.gov/geo) under accession GSE64591.

### Statistical analysis

Descriptive comparisons of study variables between cases and controls used the Chi-squared test for categorical data, and the Student's t-test for continuous data. The data were analysed in HTqPCR package [24] using R Bioconductor [25]. miRNAs with undetermined Ct values in more than 120 samples were filtered out. Data were quantile normalized. Following normalization Ct values with an interquartile range (IQR) of less than 1.5 and endogenous controls were removed from subsequent analysis, and limma analysis was performed to identify differentially regulated miRNA between cases and controls. With limma, a one-factorial linear model is fitted for each miRNA and the standard errors (SE) are moderated using an empirical Bayes model resulting in moderated *t*-statistics for each miRNA [26]. P-values of less than 0.05 were considered statistically significant. We also report adjusted p-values corrected for multiple testing using the Benjamini-Holm method to control for the false positive error rate. Logistic regression with a lasso penalty (with penalty parameter tuning conducted by 20-fold cross-validation) was used to select a panel of miRNAs for discriminating between cases and controls. Logistic regression models were used to evaluate whether the 24-miRNA panel was associated with lung cancer after adjustment for known risk factors for lung cancer (age, sex and smoking status). The area under the receiver operating characteristic curve (AUC) was calculated to assess the discriminatory power of the model. Internal validation of the selected model was conducted by calculating the bootstrap optimism-corrected AUC. This correction accounts for overfitting of the model parameters for the selected model. Additionally, an optimism-correct AUC was calculated based on bootstrapping the entire model selection process. This accounts for overfitting in both model selection and parameter estimation. All analyses were performed by using STATA v11 (STATA, College Station, TX) and R [25]. All presented p-values are two-sided.

## Supporting Information

S1 Fig Hierarchical classification of 61 significant differentially expressed microRNAs (p-value < 0.05) in lung cancer patients as compared with controls. NSCLC cases are highlighted in red.
(TIF)

S2 Fig Optimal lambda value and cross validated error plot for the Lasso logistic regression analysis. (TIF)

S3 Fig Box plots of the 24 plasma miRNAs included in the panel in lung cancer patients and controls in the IARC case-control study (2006–2012). The median score is the line in the middle of the box and the 25^th^ and 75^th^ percentile are the lower and upper part of the box. The whiskers extend to the most extreme point no longer than 1.5 times the interquartile range away from the box. Outliers are given as dots.
(TIF)

S4 Fig Assessment of the predictive value of previously described multi-miRNA signatures for an early detection of NSCLC patients in the IARC case-control study dataset (2006–2012). (TIF)

S1 Methods Supplementary methods. (DOCX)

S1 Table Logistic regression prediction model with the microRNA panel reported by Bianchi F *et al* (2011) [9] evaluated in the IARC case-control study (2006–2012). (DOCX)

S2 Table Logistic regression prediction model with the microRNA panel reported by Chen X *et al* (2012) [13] evaluated in the IARC case-control study (2006–2012). (DOCX)

S3 Table Logistic regression prediction model with the 16-microRNA ratio signature of risk reported by Boeri M *et al* (2011) [10] evaluated in the IARC case-control study (2006–2012). (DOCX)

S4 Table Logistic regression prediction model with the 16-microRNA ratio signature of diagnosis reported by Boeri M *et al* (2011) [10] evaluated in the IARC case-control study (2006–2012). (DOCX)

S5 Table Assessment of the haemolysis-related miRNAs in lung cancer patients as compared with controls in the IARC case-control study (2006–2012). (DOCX)

## References

[1] Ferlay J, Soerjomataram I, Ervik M, Dikshit R, Eser S, Mathers C, et al (2013) GLOBOCAN 2012 v1.0, Cancer Incidence and Mortality Worldwide: IARC CancerBase No. 11 [Internet]. Lyon, France: International Agency for Research on Cancer Available: http://globocan.iarc.fr. Accessed 22 January 2014.

[2] Siegel R, Naishadham D, Jemal A (2012) Cancer statistics, 2012. CA Cancer J Clin 62: 10–29. 10.3322/caac.20138 22237781

[3] Aberle DR, Adams AM, Berg CD, Black WC, Clapp JD, Fagerstrom RM, et al (2011) Reduced lung-cancer mortality with low-dose computed tomographic screening. N Engl J Med 365: 395–409. 10.1056/NEJMoa1102873 21714641 PMC4356534

[4] Bach PB, Mirkin JN, Oliver TK, Azzoli CG, Berry DA, Brawley OW, et al (2012) Benefits and harms of CT screening for lung cancer: a systematic review. JAMA 307: 2418–2429. 10.1001/jama.2012.5521 22610500 PMC3709596

[5] Iorio MV, Croce CM (2009) MicroRNAs in cancer: small molecules with a huge impact. J Clin Oncol 27: 5848–5856. 10.1200/JCO.2009.24.0317 19884536 PMC2793003

[6] miRBase (2014) miRBase: the microRNA database. Available: http://www.mirbase.org/. Accessed 23 January 2014.

[7] Chen X, Ba Y, Ma L, Cai X, Yin Y, Wang K, et al (2008) Characterization of microRNAs in serum: a novel class of biomarkers for diagnosis of cancer and other diseases. Cell Res 18: 997–1006. 10.1038/cr.2008.282 18766170

[8] Mitchell PS, Parkin RK, Kroh EM, Fritz BR, Wyman SK, Pogosova-Agadjanyan EL, et al (2008) Circulating microRNAs as stable blood-based markers for cancer detection. Proc Natl Acad Sci U S A 105: 10513–10518. 10.1073/pnas.0804549105 18663219 PMC2492472

[9] Bianchi F, Nicassio F, Marzi M, Belloni E, Dall'olio V, Bernard L, et al (2011) A serum circulating miRNA diagnostic test to identify asymptomatic high-risk individuals with early stage lung cancer. EMBO Mol Med 3: 495–503. 10.1002/emmm.201100154 21744498 PMC3377091

[10] Boeri M, Verri C, Conte D, Roz L, Modena P, Facchinetti F, et al (2011) MicroRNA signatures in tissues and plasma predict development and prognosis of computed tomography detected lung cancer. Proc Natl Acad Sci U S A 108: 3713–3718. 10.1073/pnas.1100048108 21300873 PMC3048155

[11] Sozzi G, Boeri M, Rossi M, Verri C, Suatoni P, Bravi F, et al. (2014) Clinical Utility of a Plasma-Based miRNA Signature Classifier Within Computed Tomography Lung Cancer Screening: A Correlative MILD Trial Study. J Clin Oncol. 10.1200/JCO.2013.50.4357 PMC4876348 24419137

[12] Wang K, Yuan Y, Cho JH, McClarty S, Baxter D, Galas DJ (2012) Comparing the MicroRNA spectrum between serum and plasma. PLoS One 7: e41561 10.1371/journal.pone.0041561 22859996 PMC3409228

[13] Chen X, Hu Z, Wang W, Ba Y, Ma L, Zhang C, et al (2012) Identification of ten serum microRNAs from a genome-wide serum microRNA expression profile as novel noninvasive biomarkers for nonsmall cell lung cancer diagnosis. Int J Cancer 130: 1620–1628. 10.1002/ijc.26177 21557218

[14] Heegaard NH, Schetter AJ, Welsh JA, Yoneda M, Bowman ED, Harris CC (2012) Circulating micro-RNA expression profiles in early stage nonsmall cell lung cancer. Int J Cancer 130: 1378–1386. 10.1002/ijc.26153 21544802 PMC3259258

[15] Hu Z, Chen X, Zhao Y, Tian T, Jin G, Shu Y, et al (2010) Serum microRNA signatures identified in a genome-wide serum microRNA expression profiling predict survival of non-small-cell lung cancer. J Clin Oncol 28: 1721–1726. 10.1200/JCO.2009.24.9342 20194856

[16] Shen J, Todd NW, Zhang H, Yu L, Lingxiao X, Mei Y, et al (2011) Plasma microRNAs as potential biomarkers for non-small-cell lung cancer. Lab Invest 91: 579–587. 10.1038/labinvest.2010.194 21116241 PMC3130190

[17] Kirschner MB, Edelman JJ, Kao SC, Vallely MP, van Zandwijk N, Reid G (2013) The Impact of Hemolysis on Cell-Free microRNA Biomarkers. Front Genet 4: 94 10.3389/fgene.2013.00094 23745127 PMC3663194

[18] Kirschner MB, van Zandwijk N, Reid G (2013) Cell-free microRNAs: potential biomarkers in need of standardized reporting. Front Genet 4: 56 10.3389/fgene.2013.00056 23626598 PMC3630323

[19] Pritchard CC, Kroh E, Wood B, Arroyo JD, Dougherty KJ, Miyaji MM, et al (2012) Blood cell origin of circulating microRNAs: a cautionary note for cancer biomarker studies. Cancer Prev Res (Phila) 5: 492–497. 10.1158/1940-6207.CAPR-11-0370 22158052 PMC4186243

[20] Frampton AE, Krell J, Gall TM, Castellano L, Stebbing J, Jiao LR (2014) miR-15b and miR-17 Are Tumor-Derived Plasma MicroRNAs Dysregulated in Colorectal Neoplasia. Ann Surg [Epub ahead of print]. 10.1097/SLA.0000000000000605 24646542

[21] Lin L, Lin Y, Jin Y, Zheng C (2013) Microarray analysis of microRNA expression in liver cancer tissues and normal control. Gene 523: 158–160. 10.1016/j.gene.2013.02.055 23583794

[22] Hennessey PT, Sanford T, Choudhary A, Mydlarz WW, Brown D, Adai AT, et al (2012) Serum microRNA biomarkers for detection of non-small cell lung cancer. PLoS One 7: e32307 10.1371/journal.pone.0032307 22389695 PMC3289652

[23] Hurley J, Roberts D, Bond A, Keys D, Chen C (2012) Stem-loop RT-qPCR for microRNA expression profiling. Methods Mol Biol 822: 33–52. 10.1007/978-1-61779-427-8_3 22144190

[24] Dvinge H, Bertone P (2009) HTqPCR: high-throughput analysis and visualization of quantitative real-time PCR data in R. Bioinformatics 25: 3325–3326. 10.1093/bioinformatics/btp578 19808880 PMC2788924

[25] Gentleman RC, Carey VJ, Bates DM, Bolstad B, Dettling M, Dudoit S, et al (2004) Bioconductor: open software development for computational biology and bioinformatics. Genome Biol 5: R80 15461798 10.1186/gb-2004-5-10-r80 PMC545600

[26] Smyth G, editor (2005) Limma: linear models for microarray data New York: Springer. 397–420 p.

