# Supplementary figures and images for: Circulating MicroRNAs as Non-Invasive Biomarkers for Early Detection of Non-Small-Cell Lung Cancer
Source: PLoS One. 2015 May 12;10(5):e0125026. doi: 10.1371/journal.pone.0125026 (PMC4428831; doi:10.1371/journal.pone.0125026)

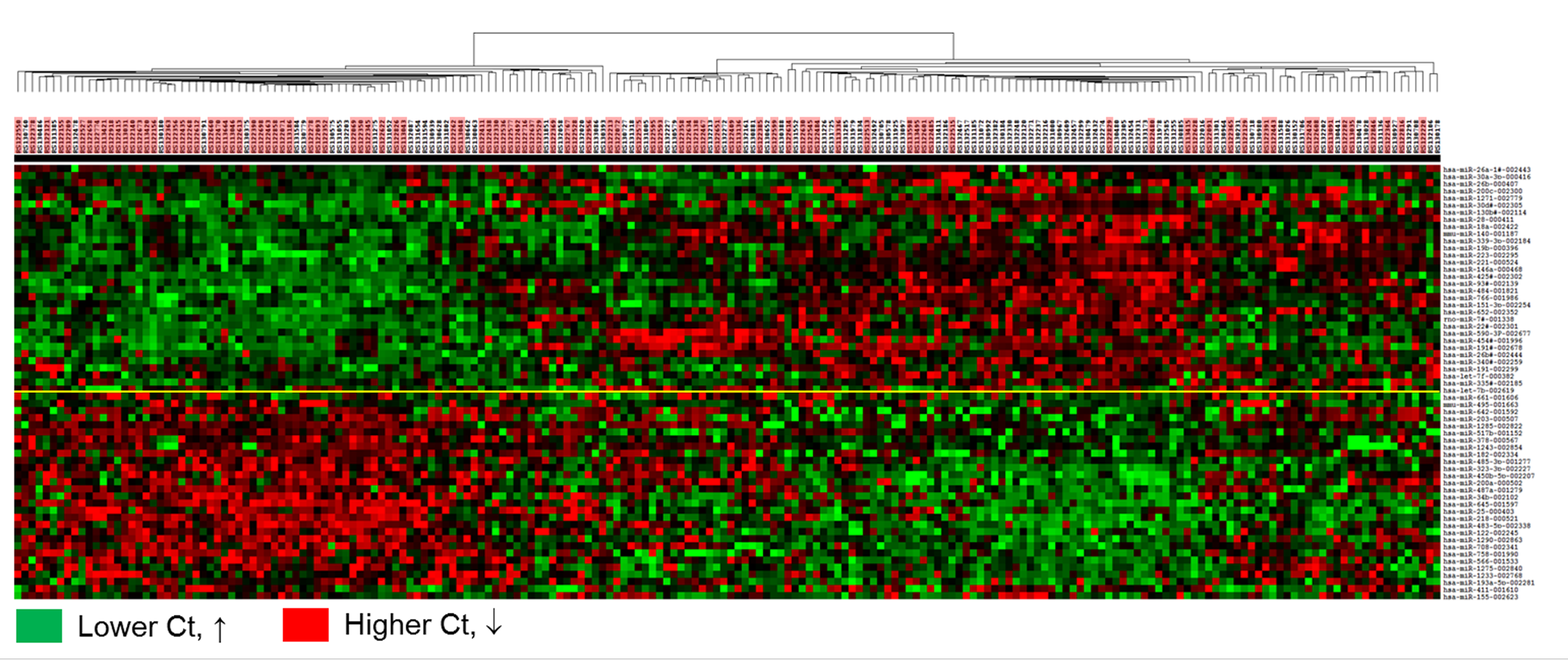

Supplement: S1 Fig — NSCLC cases are highlighted in red. (TIF) [file pone.0125026.s001.tif]

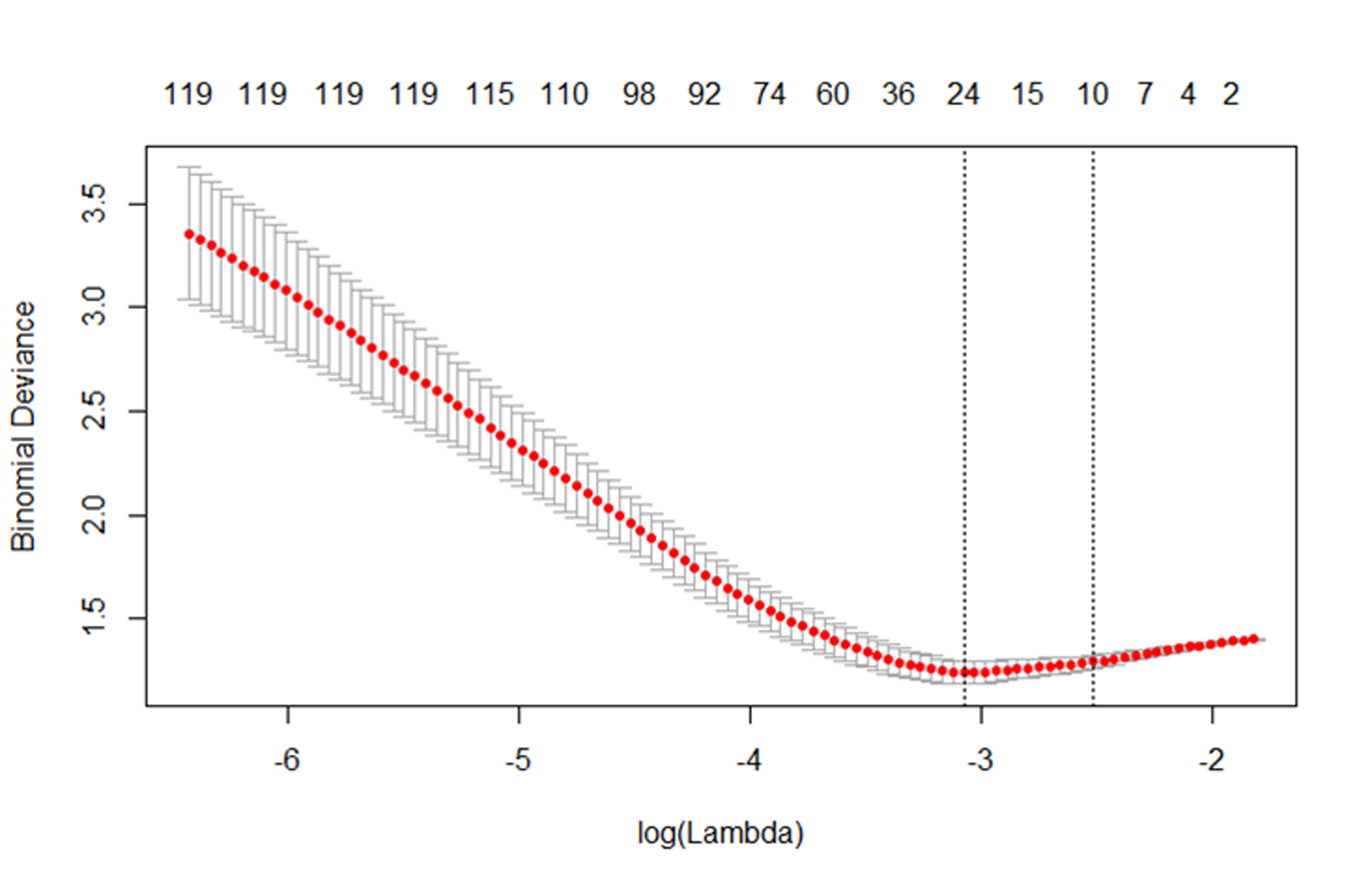

Supplement: S2 Fig — (TIF) [file pone.0125026.s002.tif]

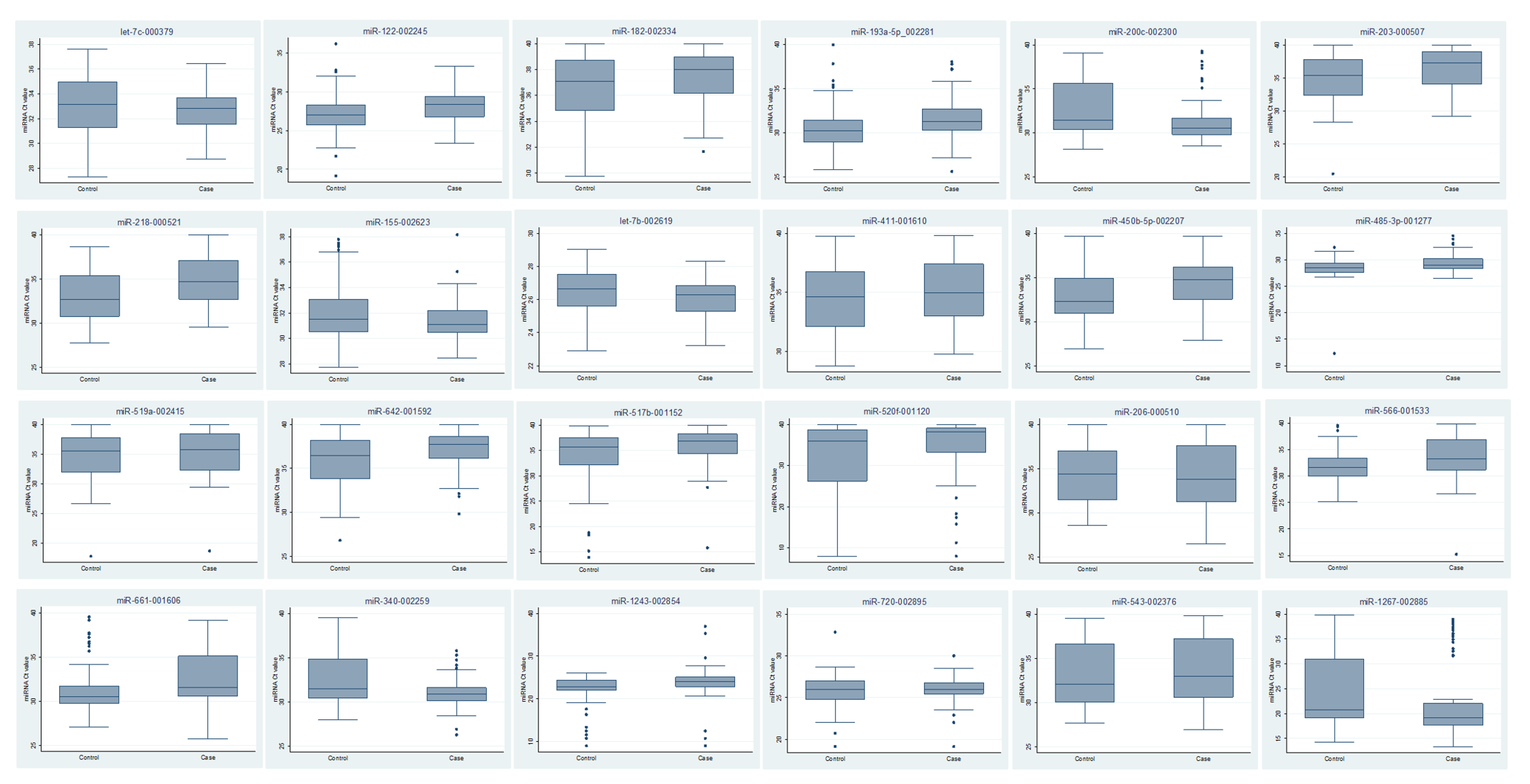

Supplement: S3 Fig — The median score is the line in the middle of the box and the 25th and 75th percentile are the lower and upper part of the box. The whiskers extend to the most extreme point no longer than 1.5 times the interquartile range away from the box. Outliers are given as dots. (TIF) [file pone.0125026.s003.tif]

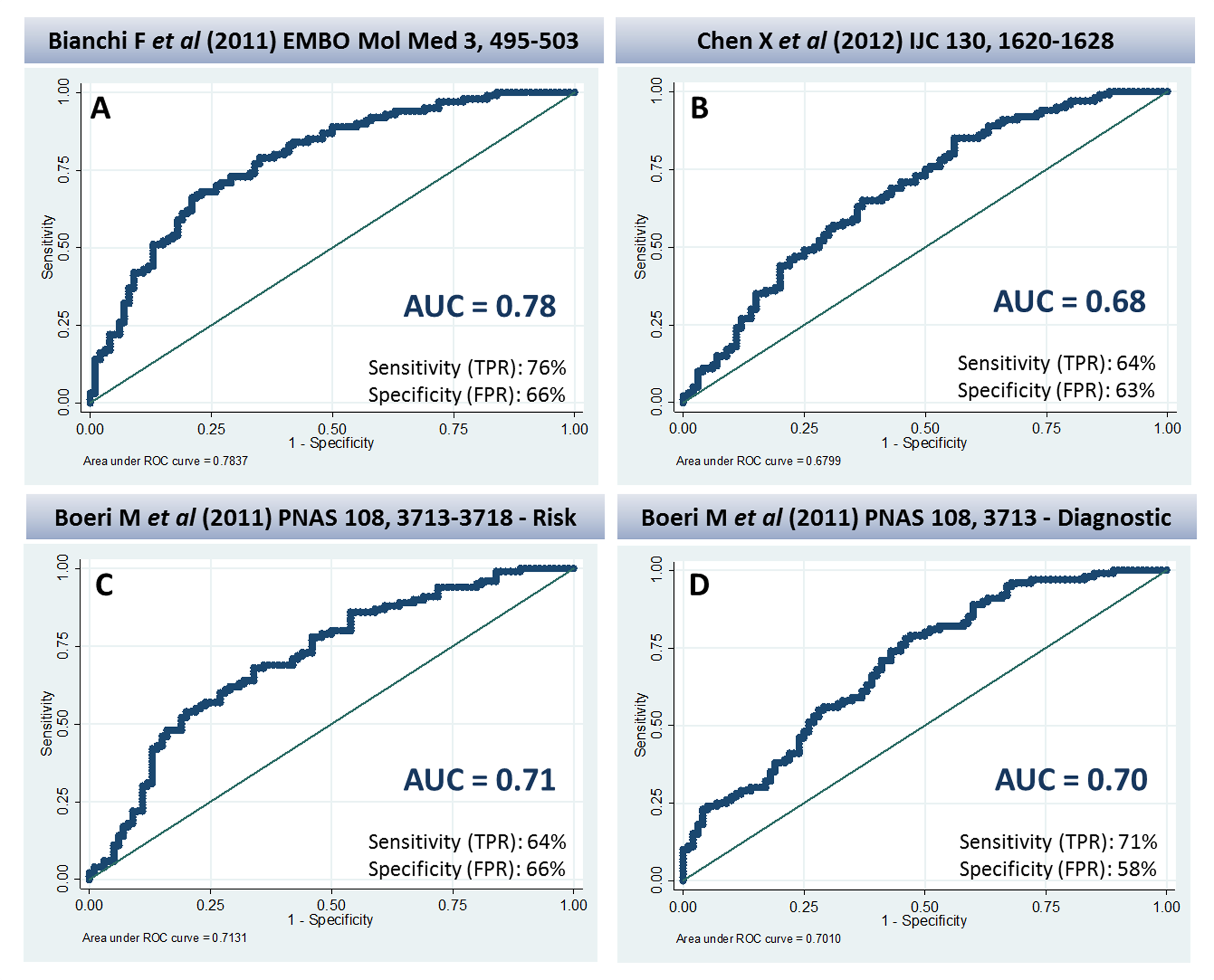

Supplement: S4 Fig — (TIF) [file pone.0125026.s004.tif]
