# Supplementary material for: Circulating MicroRNAs as Non-Invasive Biomarkers for Early Detection of Non-Small-Cell Lung Cancer
Source: PLoS One. 2015 May 12;10(5):e0125026. doi: 10.1371/journal.pone.0125026 (PMC4428831; doi:10.1371/journal.pone.0125026)
Supplement: S1 Methods — (DOCX) [file pone.0125026.s005.docx]

**S1 Methods. Online Supplementary Methods**

**Selection of cases and controls**

The selection criteria for cases included availability of tumour and plasma samples, stage (stages IA to IIIA were selected), follow-up information for at least two years (unless death occurred in before 2 years), survival time longer than 1 month after diagnosis, and diagnosis with major two NSCLC histological types (AC, SCC). Controls were randomly selected among the recruited hospital-based controls. The most frequent reasons for being at hospital were diseases of the nervous system and sense organs (22%, e.g., glaucoma, disease of the ear), followed by diseases of the digestive system (21%); diseases of the circulatory system (17%); diseases of the genitourinary system (10%); diseases of the musculoskeletal system and connective tissue (8%); injury and poisoning (8%); endocrine, nutritional and metabolic diseases and immunity disorders (7%); diseases of blood and blood-forming organs (4%); and diseases of the skin and subcutaneous tissue (3%).

**Profiling by TaqMan Human MicroRNA Arrays**

Briefly, 3 μL of total RNA (nonenriched, containing small RNA species) was reverse-transcribed into cDNA by the TaqMan MicroRNA Reverse Transcription Kit and Megaplex RT set pool A and B v3.0 (Applied Biosystems). The resulting cDNAs (2.5 μL) was then pre-amplified for 12 cycles using the corresponding Megaplex™ PreAmp Primers and TaqMan PreAmp MasterMix. Real-time quantitative PCR (RT-qPCR) was carried out with 9 μL of 1 in 4 diluted cDNA template was diluted and mixed with TaqMan Universal PCR Master Mix, and loaded into each of the eight fill ports on the TaqMan Array. TaqMan MicroRNA Arrays were then run using the ABI 7900HT Real-Time PCR platform. All reactions were performed according to standard manufacturers' protocols.
