# Supplementary material for: Circulating MicroRNAs as Non-Invasive Biomarkers for Early Detection of Non-Small-Cell Lung Cancer
Source: PLoS One. 2015 May 12;10(5):e0125026. doi: 10.1371/journal.pone.0125026 (PMC4428831; doi:10.1371/journal.pone.0125026)
Supplement: S1 Table — (DOCX) [file pone.0125026.s006.docx]

**S1 Table.** **Logistic regression prediction model with the microRNA panel reported by Bianchi F *et al* (2011) [**[**9**](#_ENREF_9)**] evaluated in the IARC case-control study (2006-2012).**

| miRNA | OR^a^ | 95% CI | P value |
| --- | --- | --- | --- |
| miR-92a-000431 | 1.05 | 0.86-1.29 | 0.618 |
| miR-484-001821 | 1.09 | 0.81-1.47 | 0.552 |
| miR-486-001278 | 0.93 | 0.77-1.12 | 0.427 |
| miR-328-000543 | 1.18 | 0.91-1.52 | 0.207 |
| miR-191-002299 | 0.91 | 0.46-1.78 | 0.777 |
| miR-376a-000565 | 0.96 | 0.73-1.27 | 0.795 |
| miR-342-3p-002260 | 0.95 | 0.70-1.28 | 0.722 |
| miR-331-000545 | 0.92 | 0.73-1.17 | 0.506 |
| miR-30c-000419 | 1.65 | 0.73-3.70 | 0.228 |
| miR-28-000411 | 0.81 | 0.64-1.02 | 0.069 |
| miR-98-000577 | 0.95 | 0.86-1.04 | 0.237 |
| miR-17-002308 | 0.82 | 0.61-1.12 | 0.220 |
| miR-26b-000407 | 0.80 | 0.58-1.12 | 0.191 |
| miR-374-000563 | 0.97 | 0.86-1.09 | 0.595 |
| miR-30b-000602 | 0.71 | 0.22-2.27 | 0.569 |
| miR-26a-000405 | 0.99 | 0.44-2.25 | 0.981 |
| miR-142-3p-000464 | 1.49 | 0.89-2.51 | 0.131 |
| miR-103-000439 | 0.73 | 0.45-1.16 | 0.183 |
| miR-126-002228 | 0.57 | 0.29-1.09 | 0.087 |
| let-7a-000377 | 1.04 | 0.90-1.19 | 0.607 |
| let-7d-002283 | 1.61 | 0.75-3.46 | 0.225 |
| let-7b-002619 | 0.57 | 0.35-0.94 | 0.027 |
| miR-32-002109 | 0.93 | 0.82-1.06 | 0.311 |
| miR-133b-002247 | 0.94 | 0.83-1.06 | 0.306 |
| miR-566-001533 | 1.13 | 1.02-1.26 | 0.023 |
| miR-432-001026 | 1.03 | 0.89-1.20 | 0.659 |
| miR-223-002295 | 1.19 | 0.86-1.64 | 0.292 |
| miR-29a-002112 | 0.69 | 0.40-1.20 | 0.188 |
| miR-148a-000470 | 1.20 | 0.90-1.60 | 0.216 |
| miR-142-5p-002248 | 1.00 | 0.80-1.26 | 0.979 |
| miR-22-000398 | 1.06 | 0.94-1.18 | 0.363 |
| miR-148b-000471 | 1.04 | 0.82-1.31 | 0.745 |
| miR-140-001187 | 0.90 | 0.65-1.24 | 0.509 |
| miR-139-5p-002289 | 1.23 | 0.68-2.21 | 0.491 |

^a^ Model containing 34-miRNA panel (continuous, normalized Ct values)

Abbreviations: OR, odds ratio; CI, confidence interval
