# Supplementary material for: Circulating MicroRNAs as Non-Invasive Biomarkers for Early Detection of Non-Small-Cell Lung Cancer
Source: PLoS One. 2015 May 12;10(5):e0125026. doi: 10.1371/journal.pone.0125026 (PMC4428831; doi:10.1371/journal.pone.0125026)
Supplement: S2 Table — (DOCX) [file pone.0125026.s007.docx]

**S2 Table.** **Logistic regression prediction model with the microRNA panel reported by Chen X *et al* (2012) [**[**13**](#_ENREF_13)**] evaluated in the IARC case-control study (2006-2012).**

| miRNA | OR^a^ | 95% CI | P value |
| --- | --- | --- | --- |
| miR-20a-000580 | 0.45 | 0.24-0.82 | 0.010 |
| miR-24-000402 | 1.08 | 0.71-1.66 | 0.722 |
| miR-25-000403 | 1.32 | 1.07-1.64 | 0.010 |
| miR-145-002278 | 0.94 | 0.77-1.15 | 0.551 |
| miR-152-000475 | 0.96 | 0.81-1.14 | 0.635 |
| miR-199a-3p-000498 | 1.12 | 0.81-1.54 | 0.509 |
| miR-221-000524 | 0.91 | 0.66-1.24 | 0.547 |
| miR-222-002276 | 1.56 | 0.83-2.93 | 0.165 |
| miR-223-002295 | 1.01 | 0.77-1.33 | 0.936 |
| miR-320-002277 | 1.23 | 0.79-1.93 | 0.363 |

^a^ Model containing 10-miRNA panel (continuous, normalized Ct values)

Abbreviations: OR, odds ratio; CI, confidence interval
