# Supplementary material for: Circulating MicroRNAs as Non-Invasive Biomarkers for Early Detection of Non-Small-Cell Lung Cancer
Source: PLoS One. 2015 May 12;10(5):e0125026. doi: 10.1371/journal.pone.0125026 (PMC4428831; doi:10.1371/journal.pone.0125026)
Supplement: S3 Table — (DOCX) [file pone.0125026.s008.docx]

**S3 Table. Logistic regression prediction model with the 16-microRNA ratio signature of risk reported by Boeri M *et al* (2011) [**[**10**](#_ENREF_10)**] evaluated in the IARC case-control study (2006-2012).**

| miRNA | OR^a^ | 95% CI | P value |
| --- | --- | --- | --- |
| miR-140-001187/miR-660-001515 | 0.56 | 0.28-1.13 | 0.106 |
| miR-140-001187/miR-320-002277 | 0.97 | 0.88-1.07 | 0.571 |
| miR-28-3p-002446/miR-660-001515 | 0.98 | 0.92-1.04 | 0.502 |
| miR-140-001187/miR-451-001141 | 0.90 | 0.49-1.64 | 0.722 |
| miR-30c-000419/miR-451-001141 | 1.25 | 0.61-2.55 | 0.542 |
| miR-140-001187/miR-92a-000431 | 1.74 | 1.07-2.83 | 0.027 |
| miR-197-000497/miR-451-001141 | 0.99 | 0.98-1.00 | 0.196 |
| miR-221-000524/miR-660-001515 | 0.92 | 0.84-1.02 | 0.101 |
| miR-19b-000396/miR-660-001515 | 0.98 | 0.86-1.11 | 0.726 |
| miR-142-3p-000464/miR-660-001515 | 2.14 | 0.98-4.69 | 0.057 |
| miR-15b-000390/miR-92a-000431 | 0.96 | 0.86-1.07 | 0.497 |
| miR-30c-000419/miR-660-001515 | 0.95 | 0.40-2.26 | 0.914 |
| miR-30b-000602/miR-92a-000431 | 0.94 | 0.78-1.13 | 0.487 |
| miR-17-002308/miR-451-001141 | 0.86 | 0.69-1.07 | 0.183 |
| miR-142-3p-000464/miR-92a-000431 | 0.57 | 0.31-1.06 | 0.077 |
| miR-106a-002169/miR-451-001141 | 1.11 | 0.88-1.39 | 0.381 |

^a^ Model containing 16-miRNA ratios (continuous, 0.01 ratio units)

Abbreviations: OR, odds ratio; CI, confidence interval
