# Supplementary material for: Circulating MicroRNAs as Non-Invasive Biomarkers for Early Detection of Non-Small-Cell Lung Cancer
Source: PLoS One. 2015 May 12;10(5):e0125026. doi: 10.1371/journal.pone.0125026 (PMC4428831; doi:10.1371/journal.pone.0125026)
Supplement: S4 Table — (DOCX) [file pone.0125026.s009.docx]

**S4 Table. Logistic regression prediction model with the 16-microRNA ratio signature of diagnosis reported by Boeri M *et al* (2011) [**[**10**](#_ENREF_10)**] evaluated in the IARC case-control study (2006-2012).**

| miRNA | OR^a^ | 95% CI | P value |
| --- | --- | --- | --- |
| miR-21-000397/miR-92a-000431 | 1.04 | 0.96-1.11 | 0.323 |
| miR-140-3p-002234/miR-17-002308 | 0.68 | 0.49-0.94 | 0.020 |
| miR-106a-002169/miR-140-3p-002234 | 0.49 | 0.26-0.92 | 0.026 |
| miR-140-001187/miR-660-001515 | 0.98 | 0.92-1.05 | 0.559 |
| miR-19b-000396/miR-660-001515 | 0.88 | 0.44-1.76 | 0.725 |
| miR-19b-000396/miR-451-001141 | 1.00 | 0.81-1.23 | 0.994 |
| miR-17-002308/miR-30c-000419 | 0.09 | 0.01-0.77 | 0.027 |
| miR-106a-002169/miR-30c-000419 | 9.43 | 1.14-77.68 | 0.037 |
| miR-19b-000396/miR-92a-000431 | 1.10 | 0.64-1.90 | 0.736 |
| miR-15b-000390/miR-92a-000431 | 1.53 | 0.61-3.87 | 0.366 |
| miR-28-3p-002446/miR-92a-000431 | 0.56 | 0.30-1.03 | 0.061 |
| miR-28-3p-002446/miR-660-001515 | 2.08 | 0.97-4.46 | 0.060 |
| miR-17-002308/miR-486-001278 | 6.82 | 0.76-61.21 | 0.086 |
| miR-15b-000390/miR-660-001515 | 0.54 | 0.17-1.74 | 0.302 |
| miR-106a-002169/miR-486-001278 | 0.15 | 0.02-1.33 | 0.088 |
| miR-17-002308/miR-451-001141 | 0.98 | 0.80-1.19 | 0.818 |

^a^ Model containing 16-miRNA ratios (continuous, 0.01 ratio units)

Abbreviations: OR, odds ratio; CI, confidence interval
