# Supplementary material for: Circulating MicroRNAs as Non-Invasive Biomarkers for Early Detection of Non-Small-Cell Lung Cancer
Source: PLoS One. 2015 May 12;10(5):e0125026. doi: 10.1371/journal.pone.0125026 (PMC4428831; doi:10.1371/journal.pone.0125026)
Supplement: S5 Table — (DOCX) [file pone.0125026.s010.docx]

**S5 Table.** **Assessment of the haemolysis-related miRNAs in lung cancer patients as compared with controls in the IARC case-control study (2006-2012).**

| miRNA | **Cases (N=100)** | | **Controls (N=100)** | | **p-value^a^** |
| --- | --- | --- | --- | --- | --- |
|  | **Mean Ct** | **95% CI** | **Mean Ct** | **95% CI** |  |
| miR_451_001141 | 25.17 | 24.77-25.57 | 25.10 | 24.75-25.45 | 0.795 |
| miR_16_000391 | 21.34 | 20.99-21.70 | 21.62 | 21.12-22.11 | 0.366 |
| miR_15b_000390 | 26.20 | 26.04-26.35 | 26.64 | 26.29-27.00 | 0.024 |
| miR_486_3p_002093 | 31.30 | 30.84-31.77 | 31.49 | 30.91-32.07 | 0.613 |
| miR_532_3p_002355 | 31.48 | 30.99-31.96 | 31.56 | 31.04-32.08 | 0.808 |
| miR_886_5p_002193 | 34.51 | 33.92-35.11 | 35.24 | 34.56-35.91 | 0.111 |
| miR_636_002088 | 24.72 | 23.84-25.60 | 25.06 | 24.01-26.10 | 0.622 |
| miR_1255B_002801 | 35.45 | 34.79-36.12 | 36.13 | 35.47-36.79 | 0.155 |
| RNU48_001006 | 37.61 | 37.04-38.18 | 37.28 | 36.70-37.85 | 0.411 |
| miR_92a_000431 | 23.38 | 22.92-23.84 | 23.21 | 22.87-23.55 | 0.553 |

^a^ p value calculated using Student’s t-test
